# Quantitation of 2‐acetyl‐1‐pyrroline in aseptic‐packaged cooked fragrant rice by HS‐SPME/GC‐MS

**DOI:** 10.1002/fsn3.879

**Published:** 2018-12-03

**Authors:** Young‐Sang Lee, Yejin Oh, Tae‐Hyung Kim, Yoo‐Hyun Cho

**Affiliations:** ^1^ Soonchunhyang University Asan Korea; ^2^ CJ Jeiljedang Corporation Seoul Korea; ^3^ SeedPia Suwon Korea

**Keywords:** 2‐acetyl‐1‐pyrroline, aseptic package, fragrant rice, standard addition

## Abstract

Aseptic‐packaged cooked rice (APCR) is a rice‐based food product with a rapidly increasing market size, and APCR made of fragrant rice (FR) has recently appeared on the market. The fragrance of FR is produced by a combination of odoriferous compounds, among which 2‐acetyl‐1‐pyrroline (2AP) has been identified as the most important contributor to overall aroma. This study describes the development of a method to quantify 2AP in FR‐based APCR using headspace solid‐phase microextraction (SPME) coupled with gas chromatography–mass spectrometry (GC‐MS). The recovery of 2AP spiked into APCR was lower than 10%, which suggests significant matrix effects and inappropriateness of external standard‐based calibration. For standard addition calibration method, up to 1,000 ng of 2AP were spiked into APCR containing 0% to 100% FR. Subsequent regression analyses of recovered peak area (*Y*) as a function of the amount of 2AP spiked (*X*) yielded highly linear calibration curves (*R* ^2^ > 0.9917) with consistent slopes (RSD = 2.7%), regardless of FR composition. *Y*‐intercepts, however, which represent the amount of 2AP in APCR without spiking, increased linearly (*R* ^2^ = 0.9898) in proportion to the composition of FR in the APCR. The amount of 2AP in APCR, determined by extrapolating the standard addition calibration curves, also increased linearly (*R* ^2^ = 0.9963) as a function of FR composition. Practicality of developed method was tested by monitoring 2AP contents in APCR under realistic storage conditions, which successfully demonstrated 38% and 60% 2AP reductions in APCR of 20% FR after 1 and 2 months of storage at 25°C, respectively. The present study demonstrates that a standard addition method, whereby up to 1,000 ng of 2AP standard is spiked into 4 g of APCR containing 5%–100% FR in a 20‐mL headspace vial followed by SPME/GC‐MS, may serve as an effective means of quantitating 2AP in fragrant rice‐based APCR.

## 1 INTRODUCTION

Traditionally, rice is the most important staple food in the Korean diet. Its consumption, however, has rapidly decreased from 142.4 kg per capita in 1986 down to 61.8 kg in 2017 (Korean Statistics Information Service, 2018). Consumption patterns have also changed from home‐cooked rice to diverse rice‐based processed foods such as noodles, cakes, snacks, and drinks (Oh et al., 2010). Among these processed foods, the popularity of aseptic‐packaged cooked rice (APCR) is rapidly increasing. In Korea, the market for APCR has increased from $7 million in 1996 to $180 million in 2015, a 9% annual increase (Chun et al., 2007). Several scientific reports have evaluated various aspects of APCR, including the major sensory attributes determining customers’ acceptance (Kwak, Kim, Lee, & Jeong, 2015), microwave reheating‐induced quality changes (Kum, Lee, Lee, & Lee, 1996), and rice variety‐dependent changes in sensory and other qualitative traits (Chun et al., 2007; Oh et al., 2010).

Fragrant rice (FR) varieties, such as Basmati and Jasmine, boast unique flavors and have been widely cultivated in India, Bangladesh, and Thailand. Interest in FR is increasing rapidly in Korea, with new varieties being optimized for the Korean climate and consumer palate. The flavor profiles of FR are produced by over 300 volatile compounds, among which 2‐acetyl‐1‐pyrroline (2AP) is considered to be the most important in determining overall aroma (Buttery, Ling, Juliano, & Turnbaugh, 1983; Wakte et al., 2017; Wei, Handoko, Pather, Methven, & Elmore, 2017). Due to its importance, intensive research has been conducted on 2AP over the last 30 years since its identification in rice. These studies have examined odor thresholds, the biosynthetic pathways of intermediates and related genes, and factors determining its concentration in rice such as variety, cultivation practices, milling parameters, and storage conditions (Routray & Rayaguru, 2018; Wakte et al., 2017; Wei et al., 2017).

Multiple extraction and instrumental detection methods have been adopted for the quantification of 2AP in rice, including purge and trap (Buttery, Turnbaugh, & Ling, 1988), steam distillation (Lin, Hsieh, & Hoff, 1990; Mahatheeranont, Keawsa‐Ard, & Dumri, 2001), solvent extraction (Bergman et al., 2000; Mahatheeranont et al., 2001), and static or dynamic headspace sampling (Sansenya, Hua, & Chumanee, 2018; Sriseadka, Wongpornchai, & Kitsawatpaiboon, 2006) combined with gas chromatography with flame ionization detector (GC‐FID) or gas chromatography–mass spectrometry (GC‐MS). Recently, solid‐phase microextraction (SPME) coupled to GC‐MS has become increasingly popular due to its simplicity, speed, and high sensitivity. This method also boasts the ability to simultaneously obtain a full profile of the volatile compounds found in rice (Grimm, Bergman, Delgado, & Bryant, 2001; Hinge, Patil, & Nadaf, 2016; Hopfer, Jodari, Negre‐Zakharov, Wylie, & Ebeler, 2016; Laguerre, Mestres, Davrieux, Ringuet, & Boulanger, 2007; Maraval et al., 2010; Poonlaphdecha et al., 2012). To date, all of the qualitative or quantitative studies related to 2AP have been conducted with unprocessed brown, white, or cooked white rice, but not with APCR. Although multiple aromatic compounds were observed from APCR, only non‐FR variety was used for APCR preparation (Sueka, Sumitani, Okiura, & Naka, 2006). APCR, especially when made with an FR variety, is a new commodity that has only recently been developed and commercialized. Therefore, to the best of our knowledge, there have been no studies on 2AP in APCR. The quantitation of 2AP as the major flavor‐providing compound in FR‐based APCR is important for quality control and for developing products with preferred flavor profiles. This report describes a method for quantitating 2AP in FR‐based APCR.

## 2 MATERIALS AND METHODS

### 2.1 Preparation of APCR

Aseptic‐packaged cooked rice for research purposes was prepared in a pilot‐scale batch and packaged under cleanroom conditions at the CJ Corporation. To prepare APCR samples containing different amounts of 2AP, fragrant white rice of the “Cheonjihyang‐1‐se” variety was mixed with non‐fragrant “Ilpum” white rice. The proportion of FR ranged from 0% to 100%. Mixed white rice samples were soaked in water for 80 min, placed in a plastic package, steam sterilized 8 times at 145°C for 5 s, and cooked by heating at 98°C for 35 min. Cooked rice samples were sealed with packaging film, cooled to room temperature, and stored at 4°C until analysis. Materials, procedures, and conditions for APCR preparation described above are exactly same with those for commercial products except for difference in its down‐sized pilot‐scale. For studies of long‐term storage effects, prepared APCRs were stored in incubators at 25°C or 35°C for up to 2 months.

### 2.2 Sample preparation

Prior to measuring 2AP levels, APCR samples were re‐heated according to commercially provided serving instructions. The packaging film was partially peeled off up to the dotted line on the packaging, and then, the product was microwaved (Model KR‐G20EW; Daewoo Electronics Co., South Korea) for 120 s at 700 W. After fully removing the packaging film, the whole package was transferred to a cutting board and divided into 16 portions (4 columns × 4 rows) with a spatula. After cooling to room temperature for 10 min, 4.0 g of cooked rice was removed and placed in a 20‐ml headspace (HS) vial for SPME extraction.

### 2.3 Standard additions for calibration

To generate standard addition calibration curves, 100 μL of authentic 2AP standard (Toronto Research Chemicals, Toronto, Canada), containing 0–4,000 ng 2AP and 1,000 ng 2,4,6‐trimethylpyridine (TMP) as an internal standard, was spiked into the APCR samples of 0%–100% FR composition in HS vials. The spiked amounts of 2AP were adjusted according to our experimental design, which is described in detail in the Results and Discussion section. HS vials containing APCR samples for SPME/GC‐MS analyses were closed tightly with magnetic screw‐thread metal caps equipped with PTFE/silicone septa. To obtain control data on matrix effects, empty HS vials (i.e., without an APCR sample) were spiked with 2AP standards. In each set of experiments, the peak areas corresponding to 2AP were normalized based on the average peak area of the internal standard. The resulting relative peak areas were used in statistical analyses. Calibration curves plotted the relative 2AP peak area (*Y*, dependent variable) as a function of the spiked amount of 2AP (*X*, independent variable). The *X*‐intercept of a given calibration curve, representing the amount of 2AP in an unspiked APCR sample, was obtained by dividing the *Y*‐intercept of the curve by the slope. The 2AP content in each APCR (in ng/g) was calculated after considering both the amount of 2AP and the total APCR sample weight in a given HS vial.

### 2.4 HS‐SPME and GC‐MS procedure

For quantitative measurements of 2AP, HS vials containing APCRs were pre‐heated for 40 min at 80°C. Any 2AP in the headspace was extracted and adsorbed onto a solid‐phase microextraction (SPME) fiber (divinylbenzene/carboxen/polydimethylsiloxane StableFlex fiber, Supleco, Bellefonte, PA, USA) for 25 min at 80°C. Adsorbed 2AP was desorbed by inserting the SPME fiber into the injection port of a GC‐MS (QP2010 Plus, Shimadzu, Japan) for 10 min. After desorption and prior to the next analysis, SPME fibers were thermally cleaned by placing in a heated chamber for 5 min at 260°C. An Rxi‐5Sil MS capillary column (30 m, 0.25 mm, Restek, USA) was used for separation, and the oven temperature was initially held at 40°C for 5 min, increased to 160°C at 2.5°C/min, then increased to 270°C at 20°C/min, and held for 5 min. The inlet port was operated in splitless mode and maintained at 250°C with a constant 1 ml/min flow of helium gas (99.9999%). The ion source and interface temperatures were 200 and 270°C, respectively. The electron impact energy was set to 70 eV, and a scan range from 35 to 220 *m/z* was applied. The 2AP and TMP peaks were identified based on the resulting sample mass spectra and comparisons with those of authentic standards and the NIST08 mass spectral library (Shimadzu, Japan). Peak areas were determined by integrating the peaks corresponding to 2AP (at *m/z* 83) and TMP (at *m/z* 121) in selected ion monitoring (SIM) mode.

### 2.5 Statistics

Analyses were performed in triplicate with each of three individual APCR packages for each experimental condition. Mean values and standard deviations from triplicate experiments, regression analyses, and analysis of variance (ANOVA) followed by mean difference tests, were performed with SPSS (IBM Corp., 2016) statistics software in accordance with Duncan's multiple range test at *p *<* *0.05.

## 3 RESULTS AND DISCUSSION

### 3.1 APCR matrix effects

**Figure 1:**
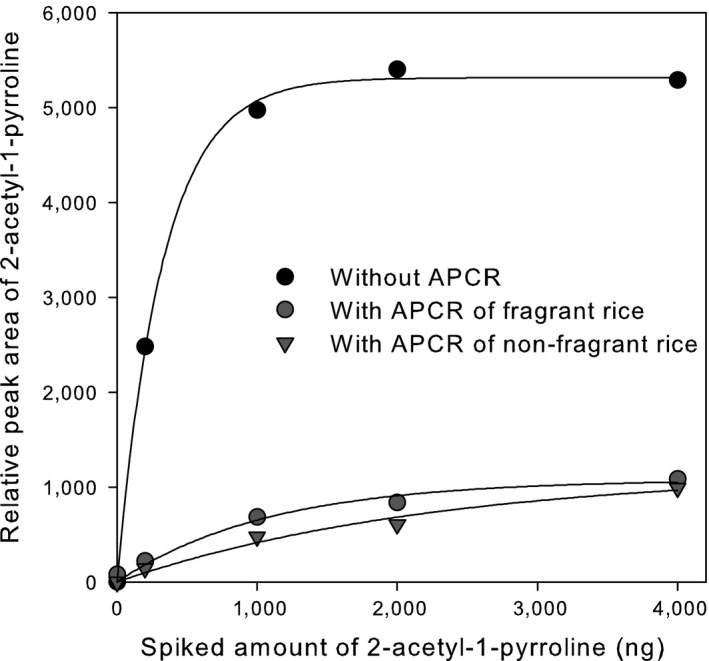
Recovered relative peak area of 2‐acetyl‐1‐pyrroline (2AP) in response to the amount of authentic 2AP standard spiked into aseptic‐packaged cooked rice (APCR) in a headspace vial. Known amounts of 2AP up to 4,000 ng were spiked into empty or APCR‐containing vials

To understand the analytical response of 2AP under our SPME/GC‐MS experimental conditions, authentic 2AP standard solutions containing 0 (blank), 200, 1,000, 2,000, or 4,000 ng of 2AP were spiked into APCR in HS vials. The recovered quantity of 2AP was evaluated by measuring the relative peak area of 2AP. APCRs were prepared with either 100% FR or 100% non‐FR. Blank controls were prepared by spiking 2AP into empty HS vials. The presence of APCR in a vial dramatically decreased the peak area of recovered 2AP. For example, spiking 200 ng of authentic 2AP standard into vials containing FR or non‐FR resulted in relative 2AP peak areas of 221 and 143, respectively, corresponding to only 8.9% and 5.8% of the peak areas observed for empty vials (2,480) spiked with the same amount (200 ng) of 2AP (Figure 1). These results suggest strong partitioning of 2AP into APCR, that is, over 90% of the spiked 2AP was retained in the APCR sample and not released into the vial headspace. These results are consistent with those of Grimm et al. (2001) who reported 2AP recoveries as low as 0.3% when using a HS‐SPME method compared to the same sample subjected to solvent extraction. Similarly, low recoveries (0.7% to 3.6%) of 2AP were observed using a headspace sorptive extraction (HSSE) method (Grimm et al., 2011), the working principle of which is similar to that of SPME. These results suggest significant “matrix effects” of APCR and indicate that external standard‐based calibration methods would be inappropriate. As an alternative, standard addition methods have been suggested to compensate for sample‐dependent recovery losses (Ouyang & Pawliszyn, 2008; Sante, 2017). Standard addition methods are commonly implemented in instrumental analyses such as HPLC and GC when significant matrix effects are expected (Danzer & Currie, 1998; Kościelniak, 1999). In standard addition procedures, known levels of analyte or authentic standard are added directly to the sample being analyzed (Ouyang & Pawliszyn, 2008).

In addition to matrix effects, saturation kinetics for 2AP in APCR were observed at spiking levels greater than 1,000 ng of 2AP (Figure 1). This phenomenon is fairly common in headspace techniques (Sriseadka et al., 2006) and SPME‐based quantitative studies and is due to the kinetic capacity of an analyte partitioning into the sorbent material on an SPME fiber (Semenov, Koziel, & Pawliszyn, 2000). Under conditions of saturation kinetics, an appropriate range of 2AP spike levels must be selected such that a linear response is obtained (Danzer & Currie, 1998). Therefore, in subsequent experiments, the spiking levels of 2AP into APCR were kept below 1,000 ng. The data in Figure 1 show that, for the same concentration of spiked 2AP, FR‐based APCR samples always exhibited a higher 2AP peak area, indicating the presence of 2AP in the original sample.

### 3.2 Development of the standard addition method

**Figure 2:**
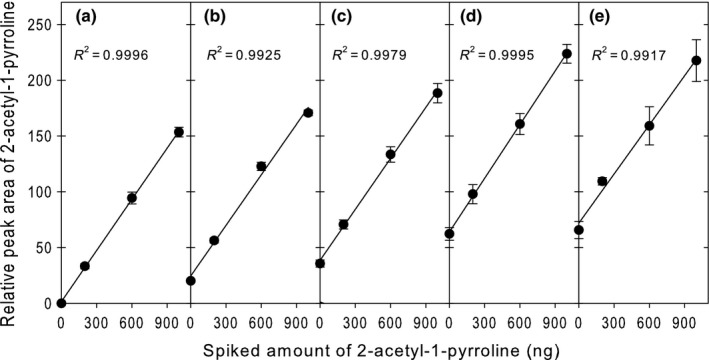
Linear increases in the recovered relative peak area of 2‐acetyl‐1‐pyrroline (2AP) as a function of the amount of authentic 2AP standard spiked into aseptic‐packaged cooked rice containing 0% (a), 25% (b), 50% (c), 75% (d), and 100% (e) fragrant rice. Data represent means ± standard deviations of three independent measurements

Aseptic‐packaged cooked rice samples containing 0, 25%, 50%, 75%, or 100% FR were spiked with 0, 200, 600, or 1,000 ng of authentic 2AP standard, and calibration curves were generated from the measured 2AP peak area as a function of the amount of spiked 2AP. In all cases, the relative peak area of recovered 2AP increased linearly in proportion to the amount of spiked 2AP (Figure 2). The resulting linearity coefficients (*R* ^2^) ranged from 0.9917 to 0.9996, indicating acceptable linearity within the tested range of 2AP concentrations. This high degree of linearity also validates the extrapolation of these calibration curves to obtain the 2AP concentrations in the initial, unspiked APCR samples.

**Figure 3:**
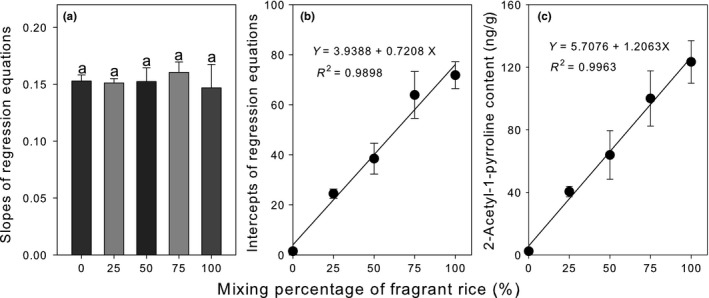
Fragrant rice composition‐dependent changes in slope (a) and *Y*‐intercept (b) of the regression equations given in Figure 2. Extrapolation of the curves in Figure 2 was used to determine the 2AP content of APCR samples as a function of the proportion of fragrant rice (c). Data represent means ± standard deviations of triplicate measurements at each proportion of fragrant rice. Same letters atop the bars in (a) indicate statistical insignificance in accordance with Duncan's multiple range test at *p *<* *0.05. APCR, aseptic‐packaged cooked rice

The slopes of calibration curves, representing the increase in recovered 2AP per unit increase in the amount of spiked 2AP amount, were consistent (RSD = 2.7%) regardless of the proportion of FR in the APCR samples (Figure 3a). These findings suggest that the relative proportions of FR and non‐FR do not influence the matrix effects of APCR on quantitative measurements of 2AP. In contrast, the *Y*‐intercepts of the calibration curves increased linearly (*R* ^2^ = 0.9898) as a function of the proportion of FR in the rice mixture (Figure 3b). This was expected because the *Y*‐intercept represents the amount of naturally occurring 2AP in the APCR sample prior to spiking.

The total amount of 2AP that was liberated from the 4‐g APCR sample was determined by extrapolation of the calibration curve. This was then used to estimate the 2AP content per unit weight of APCR. 2AP content increased linearly (*R* ^2^ = 0.9963) with the proportion of FR (Figure 3c). APCR samples containing 25%, 50%, 75%, and 100% FR yielded 2AP concentrations of 32.9, 66.0, 96.2, and 126.3 ng/g, respectively. To the best of our knowledge, this is the first report detailing the 2AP content in APCR prepared with FR.

Wide variation in the 2AP content of FR has been reported as a function of cooking status, variety, rice cultivation practices, environmental factors during cultivation, and postharvest processing (Itani, Tamaki, Hayata, Fushimi, & Hashizume, 2004; Mahmud, Oh, Kim, Cho, & Lee, 2018; Routray & Rayaguru, 2018; Wakte et al., 2017; Yoshihashi, Huong, Surojanametakul, Tungtrakul, & Varanyanond, 2005). Analysis protocols can also be a source of variations in 2AP content. Yang, Shewfelt, Lee, and Kays (2008) reported 0.2–4.5 ng/g of 2AP in cooked rice by dynamic headspace sampling, whereas continuous extraction methods employing steam distillation have yielded 40–300 ng/g of 2AP in cooked rice (Buttery et al., 1983). In preliminary tests, uncooked brown “Cheonjihyang‐1‐se” rice, the same FR variety used in this study, contained 600–1,400 ng/g of 2AP by solvent extraction method depending on storage conditions (unpublished data). These values in brown rice are around 5‐ to 11‐fold higher than those for 2AP in APCR, which suggests that a significant amount of 2AP in brown rice may be lost during APCR processing. More research, including a step‐dependent analysis of 2AP loss throughout APCR processing, is required to produce APCR that retains more 2AP, thereby maintaining higher levels of the natural flavors and aroma contained in FR varieties.

### 3.3 Practical applications of the developed method

**Figure 4:**
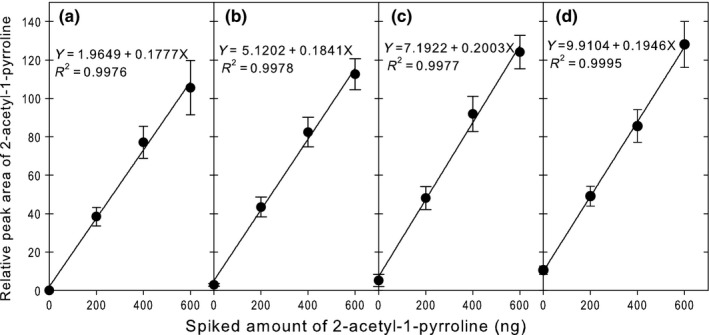
Changes in the relative peak area of 2‐acetyl‐1‐pyrroline (2AP) as a function of the amount of 2AP standard spiked into aseptic‐packaged cooked rice containing 0% (a), 5% (b), 10% (c), and 20% (d) of fragrant rice. Data represent means ± standard deviations of three independent measurements

In preliminary consumer test panels, APCR containing around 20% FR “Cheonjihyang‐1‐se” was deemed the most palatable. It is very likely that commercial APCR products contain proportions of FR much lower than 100%. In contrast to the above experiments, this portion of our study examined more industrially applicable parameters, including FR percentages from 0% to 20%, and prolonged storage conditions. Prior to examining the effects of long‐term storage on 2AP levels, the applicability of the developed method to quantitate low levels of FR in APCR was evaluated by spiking APCR samples with 0, 200, 400, and 600 ng of 2AP. As with the previous case of APCR with 0%–100% FR (Figure 2), quite similar results were observed in APCRs containing 0–20% FR (Figure 4). Calibration curves relating the recovered peak area of 2AP (*Y*) to the amount of spiked 2AP (*X*) exhibited (a) high linearity (*R* ^2^ of 0.9976 to 0.9995) and (b) consistent slopes (RSD of 5.3%, ranging from 0.1777 to 0.2003) regardless of FR composition, but (c) increasing *Y*‐intercepts in proportion to FR percentage (Figure 4). These results demonstrate (a) acceptable linearity, (b) consistent 2AP recovery efficiency that is independent of the mixing ratio of fragrant and non‐fragrant rice varieties, and (c) increasing 2AP content as a function of increasing FR composition. This method was also shown to be effective in determining 2AP levels in APCR products containing less than 20% FR.

Our preliminary tests found that 4 g of APCR contains 96 ± 5.6 kernels (mean ± *SD*,* n* = 5). Thus, 4 g of APCR made with 5% FR contains only 4 to 6 kernels of FR. From the regression equation in Figure 4b, the total amount of 2AP in such a sample would be 27.8 ng, which suggests that 5.5–6.9 ng of 2AP is released from a single kernel of cooked FR in an aseptic package. These findings are equivalent to the SPME‐based detection of 2AP in a single uncooked kernel of FR (Hopfer et al., 2016). It is therefore likely that our developed method is sensitive enough to quantitate extremely low levels of 2AP in cooked FR.

To further assess the practical uses of our developed method, typical market distribution conditions were imitated by storing APCR samples at 25°C or 35°C for up to 2 months while monitoring changes in 2AP content. At the onset of long‐term storage, APCRs containing 5%, 10%, and 20% FR contained 4.36, 8.98, and 15.33 ng/g of 2AP, respectively, once again showing increasing levels of 2AP with increasing proportions of FR (Table 1). APCRs containing 20% FR exhibited 38% and 60% reductions in 2AP content after 1 and 2 months of storage at 25°C, respectively. Higher storage temperatures, as expected, further accelerated the reduction of 2AP levels in APCR. Those made with 20% FR exhibited 50% and 66% reductions in 2AP content during the 1st and 2nd months of storage at 35°C, respectively. At 25°C, changes in the 2AP content in APCR samples containing as little as 5% FR were successfully quantified and compared (Table 1), further demonstrating the sensitivity and applicability of our developed method. Further validation, however, is required to clarify the accuracy, precision, limit of detection (LOD), limit of quantitation (LOQ), repeatability, and reproducibility of this method.

**Table 1:** Storage‐dependent changes in the 2‐acetyl‐1‐pyrroline content (2AP; ng/g) of aseptic‐packaged cooked rice (APCR) containing 5%, 10%, and 20% fragrant rice

Storage duration (months)	25°C			35°C		
	5%	10%	20%	5%	10%	20%
0	4.36 ± 1.43a†	8.98 ± 2.28a	15.33 ± 3.67a	4.36 ± 1.43a	8.98 ± 2.28a	15.33 ± 3.67a
1	2.35 ± 0.96ab	3.76 ± 1.36b	9.46 ± 2.10b	1.04 ± 0.16b	2.40 ± 0.48b	7.74 ± 2.58b
2	1.52 ± 0.16b	3.78 ± 0.38b	6.14 ± 0.62c	1.40 ± 0.14b	2.18 ± 0.22b	5.20 ± 0.56b

APCRs were stored at 25°C or 35°C for up to 2 months. Data represent means ± standard deviations of three independent measurements, and each measurement consisted of spiking 0, 200, 400, and 600 ng of authentic 2AP standard into a headspace vial containing 4 g of APCR made with different proportions of fragrant rice.

† Different letters within a column represent significant differences in accordance with Duncan's multiple range test at *p *<* *0.05.

## 4 CONCLUSION

In this report, a standard addition method incorporating SPME/GC‐MS analysis was developed for determining the levels of 2AP in APCR samples made with 5%–100% FR. Application of developed method successfully demonstrated 2AP content changes in APCR containing 20% FR from 15.3 to 9.5 ng/g and 6.1 ng/g during the 1st and 2nd month of storage at 25°C, which proved the practicality of developed method. The method described herein, however, requires spiking with at least four different levels of standard, including a blank, for each sample to be measured. An alternative strategy employing a single spike of 2AP‐*d*_*2*_ as an internal standard may simplify this process. Considering the wide variation in the 2AP content of FR, which can depend on genetic diversity, cultivation practices, and postharvest handling conditions, the range of 2AP to be spiked for generating calibration curves should be carefully adjusted depending on the 2AP content of rice materials for APCR production.

## CONFLICT OF INTEREST

The authors declare that they do not have any conflict of interest.

## ETHICAL REVIEW

This study does not involve any human or animal testing.

## INFORMED CONSENT

Written informed consent was obtained from all study participants.
